# Das Entlassmanagement deutscher Krankenhäuser für kognitiv beeinträchtigte, ältere Menschen – ein Scoping Review

**DOI:** 10.1007/s00391-020-01732-3

**Published:** 2020-05-07

**Authors:** F. Schumacher-Schönert, D. Wucherer, A. Nikelski, S. Kreisel, H. C. Vollmar, W. Hoffmann, J. R. Thyrian

**Affiliations:** 1 AG Interventionelle Versorgungsforschung, Deutsches Zentrum für Neurodegenerative Erkrankungen e. V. (DZNE) Standort Rostock/Greifswald, Ellernholzstr. 1–2, 17487 Greifswald, Deutschland; 2 Institut für Community Medicine, Abteilung Versorgungsepidemiologie und Community Health, Universitätsmedizin Greifswald, Greifswald, Deutschland; 3 Evangelisches Klinikum Bethel gGmbH, Akademisches Lehrkrankenhaus, Universität Münster, Münster, Deutschland; 4 Abteilung für Allgemeinmedizin, Ruhr-Universität Bochum, Bochum, Deutschland

**Keywords:** Demenz, Kognitive Beeinträchtigung, Krankenhaus, Deutschland, Entlassungsmanagement, Dementia, Cognitive impairment, Hospital, Germany, Discharge management

## Abstract

**Hintergrund:**

In deutschen Krankenhäusern sind etwa 40 % aller Patienten über 65 Jahre kognitiv beeinträchtigt. Für diese ist es besonders wichtig, dass die Überleitung in die Häuslichkeit möglichst bruchlos und vollumfänglich bedarfsgerecht organisiert ist.

**Ziel der Arbeit:**

Ziel der Arbeit ist es, einen systematischen Überblick über Evidenz des Entlassmanagements (EM) bei Menschen mit kognitiven Beeinträchtigungen (MmkB) oder Demenz (MmD) zu geben. Ferner soll geprüft werden, ob sich, darauf aufbauend, die Notwendigkeit eines sektorenübergreifenden Konzeptes ergibt.

**Material und Methoden:**

Anhand eines Scoping Review wurden *n* = 102 Publikationen identifiziert, von denen *n* = 6 in die Analysen eingingen.

**Ergebnisse:**

Der Artikel gibt eine Übersicht über die aktuelle Versorgung von MmkB in deutschen Akutkrankenhäusern. Generelle Informationen zum EM im Krankenhaus wurden in 3 der 6 eingeschlossenen Arbeiten gegeben. Informationen zu einem speziellen Entlass- und Versorgungsmanagement für MmkB und MmD waren in 5 von 6 Arbeiten enthalten.

**Diskussion:**

Der Artikel illustriert bestehende Versorgungslücken von älteren MmkB an der Schnittstelle des Entlassmanagements und zeigt die Notwendigkeit neuer Versorgungsmodelle. Inwieweit diese strukturell, prozessual und systemisch in die Regelversorgung eingebettet und finanziert werden können, ist bislang offen und unerforscht.

**Zusatzmaterial online:**

Zusätzliche Informationen sind in der Online-Version dieses Artikels (10.1007/s00391-020-01732-3) enthalten.

## Abstract (en)

**Background:**

In German hospitals approximately 40% of all patients over the age of 65 years are cognitively impaired (MmkB). After discharge from hospital it is particularly important for MmkB that the transition to domestic life is organized as seamlessly and as well-coordinated as possible.

**Objective:**

The aim of the study was to determine the existing evidence on discharge management (EM) in MmkB and people with dementia (MmD). Furthermore, the study examined the necessity of an intersectoral concept for MmkB.

**Material and methods:**

Based on a scoping review a total of 102 publications were identified, of which 6 articles were finally used for the evaluation.

**Results:**

The article provides an overview of the current care of MmkB in acute care hospitals in Germany. General information on EM in hospitals was given in three of the six publications included. Information on special discharge and care management for MmkB and MmD was contained in five out of six papers.

**Discussion:**

The article illustrates significant gaps in the hospital care for older MmkB, in particular at the interface of discharge management and demonstrates the need for new care models. To what extent these new care models can be structurally, procedurally and systemically embedded in the standard care and financed, is an open and unexplored question.

## Einleitung

Einer aktuellen Studie zufolge sind in deutschen Krankenhäusern etwa 40 % aller Patienten über 65 Jahre kognitiv beeinträchtigt (z. B. an Demenz erkrankt (MmD), Menschen mit kognitiven Beeinträchtigungen [MmkB]) [5]. Die Betreuung dieser Patienten erfordert besondere Unterstützung und Flexibilität und kann eine zusätzliche Herausforderung für das Krankenhauspersonal darstellen [14, 23]. Das deutsche Gesundheitssystem steht hinsichtlich des Anspruches nach Kontinuität, Ganzheitlichkeit und Integrität im Umgang mit MmkB vor dem Hintergrund der 3 Sektorengrenzen vor großen Herausforderungen.

Für MmkB kann ein Krankenhausaufenthalt eine lebensverändernde Erfahrung sein [25]. Hauptprobleme betreffen insbesondere die Schnittstelle zwischen Krankenhaus und dem häuslichen Leben; Krankenhausentlassungen sind z. T. schwer zu terminieren, Pflegeeinrichtungen können sich dementsprechend unzureichend auf die Entlassung des Patienten vorbereiten [18]. Darüber hinaus steht nach der Entlassung aus dem Krankenhaus oft keine professionelle Kontaktperson zur Verfügung, und die medizinische Nachsorge (z. B. eine fachärztliche Anschlussbehandlung) muss von den MmkB selbst oder – sofern vorhanden – von deren pflegenden Angehörigen organisiert werden [11, 21]. Versorgungslücken können zu einer vorzeitigen Institutionalisierung führen [10], erhöhen ungeplante Wiedereinweisungen ins Krankenhaus [12] und das Sterberisiko signifikant [29]. Krankenhausentlassungen von MmkB sollten so geplant werden, dass die Rückkehr in die Häuslichkeit erleichtert wird [11, 19]. Dabei ist es besonders wichtig, die Überleitung von MmkB vom Krankenhaus in die Häuslichkeit möglichst bruchlos und bedarfsgerecht zu organisieren [2].

In den letzten Jahren wurden sehr viele Bemühungen um das Entlassmanagement (EM) deutscher Krankenhäuser unternommen; unter anderem wurden Expertenstandards definiert [28] und in 2017 wurde das EM für Krankenhäuser gesetzlich verpflichtend im SGB V § 39 Absatz 1a verankert [8]. Obwohl durch die gesetzliche Verankerung eine gewisse Homogenität hergestellt werden konnte, fehlt es übergreifend an einheitlichen Standards. Evaluierte (Begleit‑)Konzepte zum Entlassmanagement bei MmkB sind rar. Modellprojekte der Pflegeüberleitung ab 2007 liegen z. B. von Wingenfeld et al. vor [30].

### Zielsetzung

Die Arbeit soll den aktuellen Stand der Wissenschaft und die vorhandene Evidenz zum Entlassmanagement bei MmkB oder MmD darstellen. Zusätzlich werden Angaben zur Notwendigkeit eines speziellen Managements für MmkB und MmD über die Sektorengrenzen daraus hergeleitet.

## Methoden

Mithilfe eines Scoping Review wurden alle relevanten, empirischen Arbeiten und Veröffentlichungen zu Studien und Projekten von MmkB und MmD in Deutschland an der Schnittstelle vom Krankenhaus zurück in die Häuslichkeit identifiziert und eine explorative Literaturanalyse durchgeführt.

### Suchstrategie

Die Suche erfolgte vom Februar bis zum Mai 2019 in *PubMed* (Zusatzmaterial online: Anlage 1).

Als finale Suchleitwörter wurden *„(hospital OR acute care) AND (dementia OR cognitive impairment OR Alzheimer’s disease) AND (discharge OR transition) AND Germany“ *verwendet. Die Begriffe wurden in Anlehnung an das PIKO-Modell [27] definiert:

- *P*opulation – Menschen mit kognitiven Beeinträchtigungen oder mit Demenz in deutschen Krankenhäusern,
- *I*ntervention – strukturiertes Überleitungs- und/oder Entlassmanagement,
- *K*ontrollgruppe – keine,
- *O*utcome – patientenzentrierte Outcomes, Angaben zu Krankenhauswiedereinweisungen, Struktur‑, Prozess- und Ergebnisqualität.

Es wurden 102 Ergebnisse in der wissenschaftlichen Literaturdatenbank *PubMed* identifiziert und einem Titelscreening unterzogen. Dreißig Artikel wurden für das darauffolgende „abstract screening“ herangezogen. Aus Titel- und Abstract screening wurden Artikel ausgeschlossen, die bereits dem Titel nach aufgrund ihres Kernthemas nicht relevant schienen; auf dieser Stufe wurden Studien zur Grundlagenforschung (*n* = 19), zu Krebs‑, Herz‑, Kreislauf-, der Transplantations- und Adoleszenzforschung ausgeschlossen. In dem anschließenden „full text screening“ wurden Artikel mit Kernthemen zu(r) geriatrischen Patienten, demenzsensibler Pflege im Krankenhaus , kognitiv beeinträchtigten Menschen ohne Bezug auf das Entlassmanagement und Artikel mit direktem Bezug zu einem Entlassmanagement im Akutkrankenhaus herangezogen. Im weiteren Verlauf des Full text screening wurden weitere 6 Artikel ausgeschlossen, da diese sich im Kern mit den Themen der Angehörigenbelastung (*n* = 2), der Veränderung des kognitiven Status während eines rehabilitativen Aufenthaltes (*n* = 2), der Vorhersage von Demenzen mittels kognitivem Screening (*n* = 2) sowie mit einem Vergleich zwischen 2 nichtakutstationären Settings („inpatient“ vs. „day clinic treatment“; *n* = 1) auseinandersetzten.

### Ein- und Ausschlusskriterien

In die Übersichtsarbeit wurden Studien und Arbeiten eingeschlossen, die sich inhaltlich mit dem Thema des Entlassmanagements deutscher Krankenhäuser im Zusammenhang mit der Versorgung kognitiv beeinträchtigter Menschen auseinandersetzten. Dabei wurden die Arbeiten durch die Autorin in 3 Kategorien eingeordnet:

- eine Information zum Entlassmanagement im Krankenhaus ist vorhanden,
- eine Information zum Entlassmanagement im Krankenhaus unter Berücksichtigung der speziellen Pflege von MmkB und MmD wird dargestellt,
- ein Bezug zur gesetzlichen Verpflichtung eines standardisierten Entlassmanagement nach § 39 (1a) SGB V seit Oktober 2017 wurde hergestellt.

### Datenextraktion

Für jede Studie wurden die Grunddaten, einschließlich Autoren, Erscheinungsjahr und Studienregion, erfasst. Ergänzend wurden Informationen zur Studienart, zum Setting, der Studienfokus, zu den Interventions- und Outcome-Parametern und die Gütekriterien der Qualitätsbewertung erfasst.

Darüber hinaus wurden patientenzentrierte Outcomes, Interventionseffekte, Gemeinsamkeiten – Unterschiede sowie die innovativen Ansätze der Studien herausgearbeitet.

Im Rahmen des Vorgehens wurden im Verlauf der Übersichtsarbeit ergänzend und – sofern vorhanden – Informationen zu Struktur‑, Prozess- und Ergebnisqualität der Krankenhäuser und des Studienvorgehens abstrahiert.

### Datenanalyse

#### Qualitätsbewertung der eingeschlossenen Studien

Die Qualitätsbewertung der eingeschlossenen Studien erfolgte über das Qualitätskriterienblatt (Zusatzmaterial online: Anlage 2) basierend auf dem Mixed Methods Appraisal Tool (MMAT). Die Bewertung der Gütekriterien erfolgte über die Leitfragen zu Objektivität, Reliabilität und Validität. Die Übersichtsarbeit wurde insgesamt unter Beachtung des PRISMA Statement für Scoping Reviews und dessen Checkliste der Berichterstattung erstellt [15]. Neben der Autorin (F. Schumacher-Schönert) sichtete ein weiterer Gutachter (Reviewer, J.R. Thyrian) die ausgewählten Artikel und bewertete diese unabhängig von der Autorin.

## Ergebnisse

### Studienauswahl

**Figure 1:**
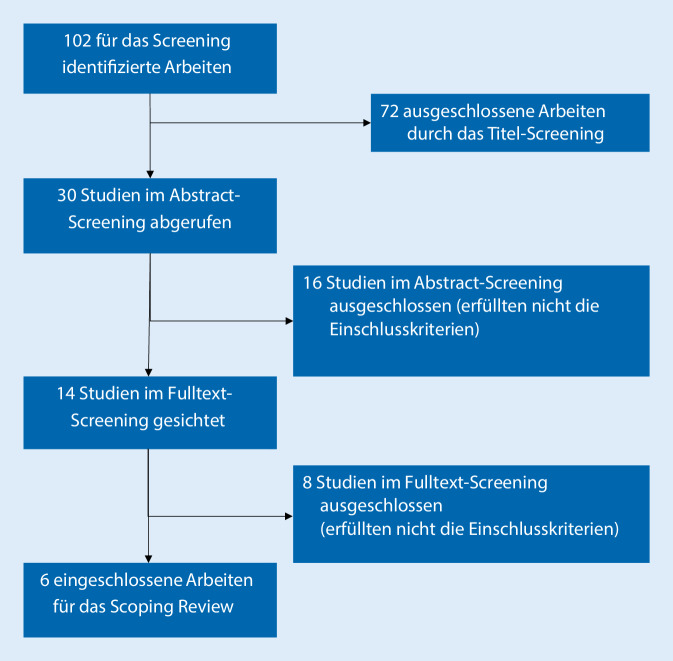

Das Flussdiagramm des Screeningprozesses ist in Abb. 1 dargestellt.

Die in Tab. 1 und 2 dargestellten 6 Artikel erfüllten die Einschlusskriterien der vorliegenden Arbeit.

**Table 1:** 

Autor (Jahr)	Titel	Quelle
Nikolaus et al. (1992)	„*Frühe Rehospitalisierung hochbetagter Patienten: Ursachen und Prävention*“	[24]
Angerhausen (2008)	„*Demenz – eine Nebendiagnose im Akutkrankenhaus oder mehr? Maßnahmen für eine bessere Versorgung demenzkranker Patienten im Krankenhaus“*	[2]
Haude et al. (2009)	„Treatment characteristics of patients with dementia: comparing two different psychiatric inpatient settings“	[17]
Bliemel et al. (2015)	*„Effect of preexisting cognitive impairment on in-patient treatment and discharge management among elderly patients with hip fractures“*	[6]
Von Renteln-Kruse et al. (2015)	„*Geriatric patients with cognitive impairment: patient characteristics and treatment results on a specialized ward“*	[26]
Zieschang et al. (2010)	*„Improving care for patients with dementia hospitalized for acute somatic illness in a specialized care unit: a feasibility study“*	[31]

**Table 2:** 

Autor (Jahr) [Ref]	Studienbewertung (max. 18)	Gütekriterien (max. 3)	Studienregion	Studientyp	Fokus	Stichprobengröße	Alter (MW in Jahren)	Diagnose	Setting	Studiendauer (in Jahren)	Bezug zu EM ab 10.2017	Info zu EM im KH	Info zu EM im KH zzgl. Demenz	Assoziierte Faktoren	Patientenzentrierte Outcomes	Interventionseffekt	Gemeinsamkeiten	Unterschiede	Innovative Ansätze	Bezug zu Struktur‑, Prozess‑, Ergebnisqualität
Nikolaus (1992) [24]	12	3	Deutschland	Retrospektive Studie	Betagte Patienten	67 (331)	81	2^b^	AKH	1987–1990	/	Teils	Teils	1^a^	k. A.	–	Idee eines ganzheitlichen Versorgungskonzeptes/-arrangements	–	–	Bezug hergestellt
Angerhausen (2008) [2]	8	2	Deutschland, NRW	Modellprojekt	Demenzpflege im Krankenhaus Auswirkungen der steigenden Zahl von Alterspatienten auf die Gesundheitspolitik und Forschung	280	k. A.	Demenz	AKH	2007–2008	/	Ja	Ja	1^a^	k. A.	Konzeptionelle Ergebnisse: Überleitung und Vernetzung, medizinische Betreuung Medizinische Betreuung: Einführung „Blauer Punkt Konsil“: MMST → verschiedene pflegerische Maßnahmen	Idee eines ganzheitlichen Versorgungskonzeptes/-arrangements	Bezieht auch Gestaltung der Räumlichkeiten im Gegensatz zu 1992 mit ein	Eher Überleitungs- als Entlassmanagement für MmD-Überleitung und -Vernetzung, medizinische Versorgung	Strukturelle und konzeptionelle Änderungsprozesse in den Anfängen
Haude et al. (2009) [17]	10	3	Deutschland	Vergleichsstudie	Verschiedene psychiatrische Ansätze im innerklinischen Setting spezialisierte Klinik während der Entlassung wurden Institutionalisierung sowie die Entlassmedikation verbessert	113	79	Demenz	DPSI	k. A.	/	/	Ja	–	Überleitung in institutionalisierte Pflege reduziert, Entlassmedikation verbessert	Interventionseffekt = Beobachtungeffekt; s. patientenzentrierte Outcomes; keine direkte Intervention	Ganzheitlicheres Versorgungskonzept vs. Routineversorgung	Patientenzentrierte Outcomes	Spezielle Demenzpflege in den Krankenhäusern wirkt positiv auf ADL und reduziert institutionalisierte Pflege	/
Zieschang et al. (2010) [31]	13	3	Deutschland	Machbarkeitsstudie, Pilotstudie	Spezialisierte Demenzpflegestation	332	82	Demenz	SCU	2004–2007	/	/	Ja	–	Barthel-Index, Mobility (Tinetti-Test): „Pre-post“-Design, „place of residence before and after the hospital stay“, „behavior“; keine Kontrollgruppe	Intervention: Aufenthalt in einer Spezialklinikabteilung, keine direkte Intervention während des Übergangs	Idee eines ganzheitlichen Versorgungskonzeptes/-arrangements; patientenzentrierte Outcomes	Wohnsituation vor und nach dem Krankenhausaufenthalt mitbetrachtet; kein gezieltes Entlassmanagement	Spezielle Demenzpflege in den Krankenhäusern wirkt positiv auf ADL	Umgebungsfaktoren: Strukturveränderungsprozesse angegeben
Bliemel et al. (2015) [6]	10	2	Deutschland	Beobachtungsstudie	Untersuchung des Einflusses einer kognitiven Beeinträchtigung auf die funktionellen Ergebnisse und die Komplikationsraten von Patienten mit Hüftfraktur während der stationären Behandlung	402	81	2^b^	AKH	2009–2011	/	Teils	Teils	5^e^	Barthel-Index,-TUG („mobility at discharge“), „destination after discharge“, „patients overall lengths“	Konzentration auf die Aufrechterhaltung der funktionellen Fähigkeiten; keine direkte Intervention	Frührehabilitation als Lösungsansatz; Entlassung in geriatrische Reha vordergründig	Perioperative Pflegetherapieziele	Perioperative Versorgung mit Erhalt der funktionellen Fähigkeiten, um Patienten vor Verlust der Unabhängigkeit und nachteiligem klinischen Verlauf zu schützen	/
Von Renteln-Kruse et al. (2015) [26]	13	3	Deutschland	Beobachtungsstudie einer Kohorte	Behandlung kognitiv beeinträchtigter Menschen im Akutkrankenhaus: Woher kommen die Patienten, wohin gehen die Patienten, welche Diagnosen, Umstände beeinflussen die Behandlung und das Ergebnis?	2084	81	2^b^	kgA	2009–2014	/	/	/	3^c^, 4^d^	ADL-BI, MMSE, Tinetti, „fall risk“, TUG, „discharge“ (Medikation etc.)	Keine direkte Intervention „because of limited resources, the patients further course after discharge was not documented in this study; nor was the perspective of relatives caring for the patients“	Gemeinsamkeiten in den patientenzentrierten Outcomes zur Frührehabilitation, aber keine konkreten Ansätze	Keinen richtigen Bezug zu EM, E,S,P-Qualität	Berücksichtigt die Wohnsituation	Bezug hergestellt

*AKH* Akutkrankenhaus, *SCU* „special care unit“, *DPSI* „different psychiatric inpatient settings“, *kgA* kognitiv geriatrische Abteilung

^a^Strukturelle und konzeptionelle Änderungsprozesse in den AKH

^b^Teils kognitiv beeinträchtigte Patienten (mit Neigung zur Multimorbidität)

^c^49,5 % Krankenhausaufnahme via Notaufnahme

^d^70 % der Patienten wurden in das vorherige Wohnumfeld zurückentlassen

^e^Funktionaler Status

^f^Pkte. 2 und 3: Die Studie erfüllt die Qualitätskriterien

### Bezug zum Entlassmanagement

Eine Information zum generellen Entlassmanagement im Krankenhaus wurde in 3 der 6 eingeschlossenen Arbeiten gegeben, Informationen zum speziellen Entlassmanagement für MmkB und MmD waren in 5 von 6 Arbeiten enthalten. Alle eingeschlossenen Arbeiten erschienen mindestens 2 Jahre vor der gesetzlichen Verpflichtung eines standardisierten Entlassmanagement seit Oktober 2017 und konnten somit keinen Bezug dazu darstellen.

Angerhausen [2] fokussierte das prozessuale, ganzheitliche Denken und Handeln im interdisziplinären Arbeitsprozess des Entlassmanagements. Im Rahmen des Entlassmanagements wurde vielfach das Medikationsmanagement und/oder die Verringerung der Überleitung in eine institutionalisierte Pflege verstanden.

### Patientenzentrierte Outcomes

**Figure 2:**
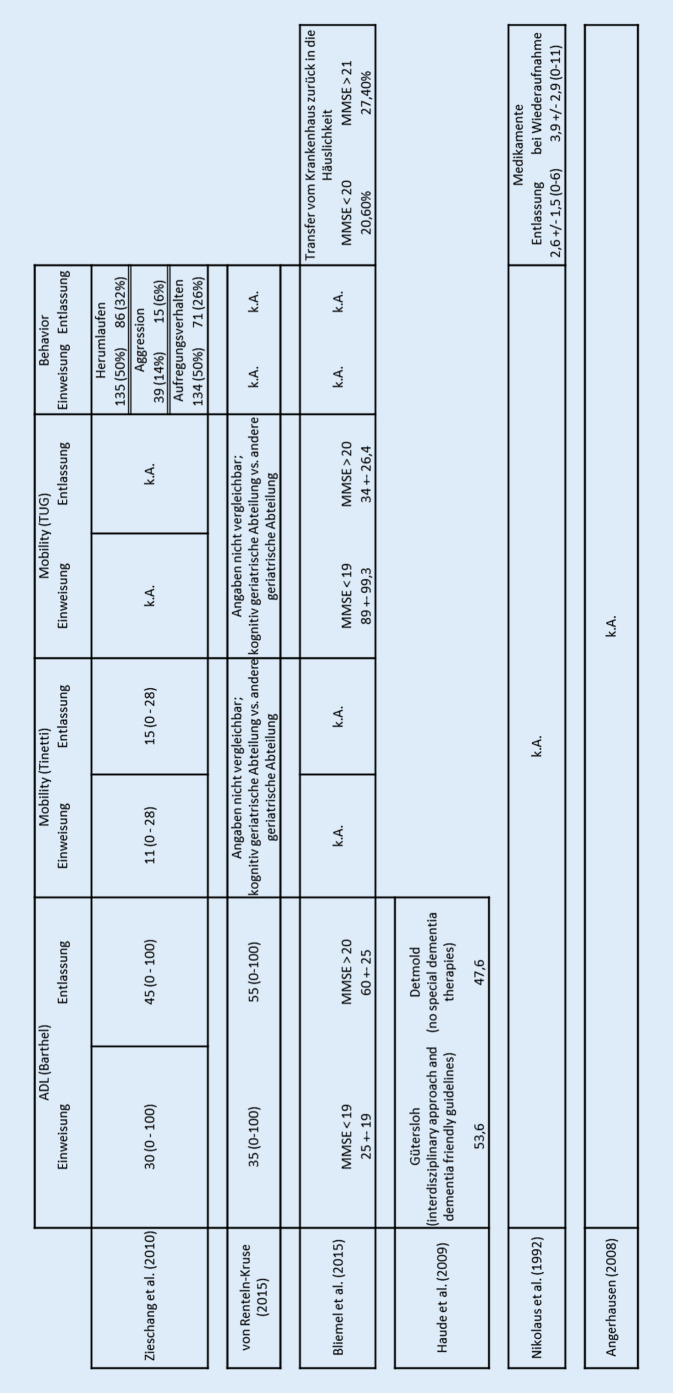

Patientenzentrierte Outcomes wurden über den funktionellen Status der Probanden (Barthel-Index), Mobilitätstest (TUG, Tinetti-Test), die Entlassmedikation und den Wohnort vor und nach dem Krankenhausaufenthalt gemessen. In den untersuchten Studien zeigte sich mehrheitlich eine Verbesserung der patientenzentrierten Outcomes, wenn ein Überleitungs- und/oder Konzept zum Umgang mit kognitiv beeinträchtigten Patienten während der Entlassung aus dem Krankenhaus zur Verfügung stand. Die Effekte eines Überleitungskonzeptes auf die patientenzentrierten Outcomes zeigt Abb. 2.

### (Interventions‑)Effekte

Die relevanten Publikationen können nach Beobachtungs- und Interventionsstudien unterschieden werden. Während es sich bei den Studien von Haude et al. [17] und Renteln-Kruse et al. [26] um reine Beobachtungen mit Gegenüberstellung der patientenzentrierten Outcomes handelt, wird in der Studie von Zieschang et al. [31] eine Interventionsgruppe (mit Aufenthalt auf einer speziellen Demenzstation) mit Daten der Gesamtklinik aus der Routinebehandlung verglichen. Bliemel et al. [6] stellen den funktionalen Status von kognitiv beeinträchtigten Patienten mit einer Schenkelhalsfraktur in den Vordergrund und kristallisieren die Frührehabilitation als Lösungsansatz für diese spezielle Patientenklientel heraus. Zwei frühere Studien von 1992 [24] und 2008 [2] geben über die patientenzentrierten Outcomes hinaus konkrete Kausalitätseffekte an. Während bei Nikolaus et al. [24] durch eine retrospektive Studie Kausalitätseffekte zur Verringerung der Rehospitalisierung aufgezeigt werden, werden im Rahmen eines ganzheitlichen Versorgungskonzeptes in der Arbeit von Angerhausen [2] konzeptionelle Effekte in den Dimensionen „Überleitung und Vernetzung“ und „medizinische Betreuung“ dargestellt. Unter anderem begleitet dabei eine Demenzbeauftragte die Patienten und deren Angehörige, schult das Personal entsprechend und unterstützt in herausfordernden Situationen im innerklinischen Setting. Weitere Orientierungshilfen im Krankenhaus und ein dicht am Alltag des Patienten strukturierter Tag (z. B. mit Berücksichtigung der Essensvorlieben etc.) helfen u. a. dabei, die Übergänge in der Versorgung möglichst reibungslos verlaufen zu lassen und Rehospitalisierungen zu verringern [2].

### Gemeinsamkeiten – Unterschiede

Zwei der 6 Studien stellen die Frührehabilitation als Lösungsansatz in den Vordergrund [6, 26]. Die Studien von Renteln-Kruse et al. und Bliemel et al. sind im Jahre 2015 erschienen. Die früheren Studien (2010, 2009, 2008, 1992) fokussieren die Idee eines ganzheitlichen, intersektoralen Versorgungsarrangements für MmkB in einem Akutkrankenhaus. Nur Angerhausen (2008) spricht die Gestaltung der Räumlichkeiten auf einer Spezialstation für MmkB an [2]. Einzig in der Studie von Bliemel [6] wurden bereits perioperative Therapieziele (z. B. Verbesserung der Mobilität während des Krankenhausaufenthaltes) benannt. Nikolaus [24] stellt retrospektiv heraus, dass die häufigsten offenen Versorgungsbedarfe und Hauptrisiken für eine frühe Rehospitalisierung in den Bereichen der Medikamenteneinnahme, des Essverhaltens (Diäten etc.) und der Hilfsmittelbereitstellung bestehen.

### Innovative Ansätze

Beide Studien aus 2015 berücksichtigen die Wohnsituation vor und nach einem akut-stationären Aufenthalt von MmkB [6, 26]. Dass eine spezielle, demenzsensible Pflege – von den Autoren als Demenzpflege beschrieben – in den Krankenhäusern den funktionellen Status (ADL) der Patienten positiv beeinflusst und die institutionalisierte Pflege nach Entlassung in die Häuslichkeit reduzieren kann, zeigten die Studien von Haude et al. und Zieschang et al. [17, 31]. Die früheren Studien betonen im Rahmen ihrer Ganzheitlichkeitsbetrachtung, dass für MmkB in einem Akutkrankenhaus eher ein Überleitungs-, denn ein Entlassmanagement zur Verfügung stehen sollte und untergliedern ein sog. Versorgungsarrangement in die Dimensionen der „medizinischen Versorgung“ und „Überleitung und Vernetzung“ [2, 24]. Wegweisend ist dabei der verknüpfende, intersektorale Ansatz durch Fokussierung auf eine vernetzende Kommunikation der behandelnden und an der Patientenbetreuung und -versorgung teilnehmenden Professionen.

### Struktur‑, Prozess- und Ergebnisqualität

Im Verlauf des Abstract screening wurden Artikel analysiert und in die Übersichtsarbeit eingeschlossen, die Angaben zu Struktur‑, Prozess- und Ergebnisqualität beinhalteten. Angaben dieser Art sind wichtige Indikatoren dafür, dass ein ganzheitliches Entlass‑/Überleitungsmanagement an der akutstationären Schnittstelle zurück in die Häuslichkeit der kognitiv beeinträchtigten Menschen perspektivisch in eine Art Regelversorgung übernommen werden kann. In 3 der 6 Studien wurden keine Angaben zu Struktur‑, Prozess- und Ergebnisqualität gemacht. Angerhausen (2008) betonte, dass sich strukturelle und konzeptionelle Änderungsprozesse sich in den Anfängen befinden [2]; Zieschang bezog Umgebungsfaktoren im Rahmen der Strukturveränderungsprozesse unter Angabe unter dem Aspekt der Kosten ein [31]. Von Renteln-Kruse et al. stellen im Zusammenhang mit ihrer Studie in einem Zitat der WHO heraus, dass „[…] auch Verbesserungen in der Struktur erforderlich [sind]“, die nicht nur die Logistik und Organisation der Pflege betreffen. „Diese Aufgaben liegen in der Verantwortung aller beteiligten Fachkräfte […]“ [26].

## Diskussion

Zusammenfassend konnten kaum Publikationen, die das Entlassmanagement deutscher Krankenhäuser im Umgang mit kognitiv beeinträchtigten Menschen thematisieren und der internationalen, wissenschaftlichen Literatur zugänglich sind, gefunden werden. Untersuchungen zu Interventionsstudien, die ein reines Übergangsmanagement von MmkB an der Schnittstelle Akutkrankenhaus – Häuslichkeit eruieren, fanden nicht statt. Patientenzentrierte Outcomes an der Schnittstelle Klinik – Häuslichkeit geben Anhaltspunkte für Interventionsmöglichkeiten. Diese bieten jedoch kein evidenzbasiertes Feedback, welches Entscheidungsträger zu einem Umdenken in der Versorgungslandschaft und -qualität an der Schnittstelle Akutkrankenhaus – Häuslichkeit von Menschen mit kognitiv beeinträchtigten Menschen leiten könnte. Diese sind jedoch wichtig, weil z. B. in den letzten Jahren in deutschen Krankenhäusern zahlreiche geriatrische Fachabteilungen gegründet wurden [22]. Die Altersmedizin verfügt mittlerweile über die zweitgrößte Anzahl von spezialisierten internistischen Betten in deutschen Krankenhäusern (Stand 09.2019). Die Studien, die dieser Analyse zugrunde liegen, geben Hinweise, dass „die frühzeitige und kontinuierliche Einbindung geriatrischer Kompetenz in die Behandlungsabläufe die Qualität der Versorgung hochbetagter, multimorbider Patienten steigern“ kann; inwiefern hiervon ein Entlassmanagement profitiert, bedarf weiterer, zielgerichteter Untersuchungen (www.dggeriatrie.de, Stand 09.2019 [32]).

Es ist jedoch unklar, ob das Entlassmanagement kognitiv beeinträchtigter Menschen durch das Vorhandensein oder die Etablierung einer geriatrischen Abteilung in einem Akutkrankenhaus bereits ausreichend verbessert wird. Neben der Gründung von Fachabteilungen wird aber auch zunehmend konzeptionell im Akutkrankenhaus den Bedarfen der MmkB Rechnung getragen. Unter dem Begriff des demenzsensiblen Krankenhauses finden sich Bestrebungen und Beispiele, wie ein multiprofessionelles Versorgungsmanagement umgesetzt werden kann [1, 20].

### Limitationen

Bei diesem Scoping Review handelt es sich um eine Übersichtsarbeit, in die Publikationen der internationalen, wissenschaftlichen Literaturdatenbank *PubMed* eingingen. Es wurden nur Artikel herangezogen, dessen Erhebungsort die Bundesrepublik Deutschland war.

Die Arbeit ist durch eine hohe externe Validität unter Berücksichtigung und der Prüfung einschlägiger Qualitätskriterien gekennzeichnet.

### Einschränkungen, Herausforderungen und zukünftige Forschung

Es gibt Hinweise, dass das sektorenübergreifende Entlassmanagement im Allgemeinkrankenhaus als Teil der akutstationären Versorgung eine breite Akzeptanz gefunden habe [16], eine solide empirische Evidenz fehlt jedoch. Welche Konzepte eine bessere und effizientere und auf das Behandlungsergebnis kognitiv beeinträchtigter Patienten noch stärker patientenzentrierte Versorgung fördern, bleibt unklar. Es existieren Veröffentlichungen um den Themenkomplex „Entlassmanagement, Überleitungsmanagement, Pflegeüberleitung“ [3, 4, 7, 9, 13]. Ein effektives (in diesem Sinne *demenzsensibles*) Entlassmanagement erfordert ein komplexes, multiprofessionelles (Versorgungs‑)Management, in das zahlreiche Variablen hineinspielen, die meist keine statischen, sondern sich gegenseitig beeinflussende, dynamische Faktoren sind.

### Schlussfolgerungen

In den untersuchten Studien zeigt sich, dass frühe Rehospitalisierungen und ungeplante Institutionalisierungen sich häufig vermeiden lassen; so wird geschätzt, dass 55,5 % der Rehospitalisierungen, die durch eine inadäquate häusliche Versorgung bedingt waren, hätten vermieden werden können, wenn vor der Entlassung aus der Klinik eine individuelle [geriatrische] Gesamteinschätzung vorgenommen und das weitere Vorgehen sorgfältig geplant und aktiv vorbereitet wird [21]. Darüber hinaus wurde deutlich, dass

A. evidenzbasierte Konzepte zum Entlassmanagement bei kognitiv beeinträchtigten Patienten fehlen; diese treffen nach der Krankenhausentlassung in ihrer Häuslichkeit oftmals noch auf eine unzureichende häusliche Betreuung.
B. auf eine Demenz/kognitive Beeinträchtigung zugeschnittene Behandlungsprogramme positiven Einfluss auf die patientenzentrierten Outcomes nehmen. Es wird angenommen, dass beispielsweise Komplikationen aus einer raschen Progression der Grundkrankheit sowie eine oftmals vorliegende Noncompliance bei der Medikamenteneinnahme oder der häuslichen Ernährung resultieren [21].
C. davon auszugehen ist, dass sich mit einer umfassenden Entlassplanung bzw. einer Übergangsbetreuung nach der Entlassung, einer ausführlichen schriftlichen Dokumentation an die versorgenden Ärzte und die Unterstützung durch ambulante Dienste Rehospitalisierungen und ungeplante Heimeinweisungen reduzieren lassen.

## Fazit für die Praxis

- Auf das Krankenhaus beschränkte Konzepte zeigen positive Auswirkungen auf die patientenzentrierten Outcomes.
- Wichtige Komponenten sind: ein positiver Einfluss auf den funktionellen Status (z. B. ADL) der Patienten, eine umfassende Entlassplanung/Übergangsbetreuung, ausführliche, schriftliche Dokumentationen an die (weiter-)versorgenden Ärzte und Unterstützungsangebote durch ambulante Dienste.
- Lösungsansätze zum Management der Überleitung vom Krankenhaus zurück in die Häuslichkeit von Menschen mit kognitiven Beeinträchtigungen sollten in der Praxis erprobt, evaluiert und implementiert werden.

## Caption Electronic Supplementary Material




