# Supplementary material for: Discharge management in German hospitals for cognitively impaired, older people—a scoping review
Source: Z Gerontol Geriatr. 2020 May 7;54(7):695–703. [Article in German] doi: 10.1007/s00391-020-01732-3 (PMC8551109; doi:10.1007/s00391-020-01732-3)
Supplement: Supplementary file 1 [file 391_2020_1732_MOESM1_ESM.docx]

**Anlage 1 –** PubMed Suchhistorie und finale Suchstrategie

Datenbasis: PubMed Februar –Mai 2019

| ***Datenbank*** | ***Suchkriterien + -filter*** | ***Summe der Ergebnisse*** | ***Datum*** |
| --- | --- | --- | --- |
| PubMed | (((dementia) AND hospital) AND germany) AND discharge | 40 | 19.02.2019 |
| PubMed | ((("dementia"[MeSH Terms] OR "dementia"[All Fields]) AND ("hospitals"[MeSH Terms] OR "hospitals"[All Fields] OR "hospital"[All Fields])) AND ("germany"[MeSH Terms] OR "germany"[All Fields])) AND ("patient discharge"[MeSH Terms] OR ("patient"[All Fields] AND "discharge"[All Fields]) OR "patient discharge"[All Fields] OR "discharge"[All Fields]) AND ("2017/01/01"[PDAT] : "2019/12/31"[PDAT]) 19.02.2019 | 16 | 19.02.2019 |
| PubMed | ((dementia hospital germany AND ( hasabstract[text] AND free full text[sb] ) AND "last 5 years"[PDat])) AND (dementia hospital germany AND ( hasabstract[text] AND free full text[sb] ) AND "last 5 years"[PDat]) | 761 | 26.02.2019 |
| PubMed | ((((((((cognitive impairment) OR dementia) AND hospital) OR acute care) AND discharge) OR transition) AND germany AND primary care | 369 | 06.03.2019 |
| PubMed | ((((((((cognitive impairment) OR dementia) AND hospital) OR acute care) AND discharge) OR transition) AND germany AND primary care | 349 | 07.03.2019 |
| PubMed | ((hospital or acute care)) AND (dementia or cognitive impairment) AND (discharge OR transition) AND germany | 98 | 17.04.2019 |
| PubMed | (hospital OR acute care) AND (dementia OR cognitive impairment OR alzheimer´s disease) AND (discharge OR transition) AND germany | 102 | 21.05.2019 |

**Anlage 2 –** Qualitätskriterienblatt

| **author (Year):** |  | | | |  |  |  |  |
| --- | --- | --- | --- | --- | --- | --- | --- | --- |
| **peer reviewed:** | yes  no | | | |  |  |  |  |
| **points** | **2** | **1** | | **0** | **Objektivität** | **Reliabilität** | **Validität** | **Publikations-verzerrung** |
| 1. research question |  |  | |  |  |  |  |  |
| 1. sample size |  |  | |  |  |  |  |  |
| 1. population |  |  | |  |  |  |  |  |
| 1. follow-up time: |  |  | |  |  |  |  |  |
| 1. follow-up rate |  |  | |  |  |  |  |  |
| 1. dropout-analysis/ loss to follow-up: |  |  | |  |  |  |  |  |
| 1. selection bias minimized |  |  | |  |  |  |  |  |
| 1. response rate |  |  | |  |  |  |  |  |
| 1. comparison group |  |  | |  |  |  |  |  |
| **Overall quality rating of the paper:**  **(points of all criteria 1-9 from above)** |  | | | |  |  |  |  |
| **clearly stated research question**:  2 clearly stated  0 not clearly stated | | | **sample size:**  2 n ≥ 400 or power-analysis performed 1 n = 200-399 0 n = 0-199 | | | | |  |
| **population** 2 hoch betagte Menschen mit kognitiver Beeinträchtigung  1 Menschen mit kognitiver Beeinträchtigung 0 betagte Menschen | | | **follow-up time:**  2 ≥ 12 months or 30-day readmission-rate  1 ≥ 6 months, < 12 months  0 < 6 months | | | | |  |
| **follow-up rate** 2 ≥ 80% 1 ≥ 60% ≤ 80% 0 < 60%, not listed | | | **dropout analysis:**  2 dropout analysis performed or dropout-rate ≤ 20%  1 dropouts and reasons listed  0 no reasons for dropouts listed | | | | |  |
| **Selection bias** 2 recruited in a way to minimize selection bias 0 not recruited in a way to minimize selection bias | | | **response rate** 2 ≥ 60% 0 < 60%, not listed | | | | |  |
| **comparison group** (patients without cognitive impairment) 2 available and participants are comparable (baseline analysis/randomization check) 1 available but not comparable 0 not available | | |  |  |  |  |  |  |

**Objektivität**: die Unabhängigkeit der Versuchsergebnisse von den Rahmenbedingungen und verfälschenden Drittfaktoren (-1, 0, +1)

**Reliabilität:** die formale Genauigkeit bzw. Verlässlichkeit wissenschaftlicher Messungen (-1, 0, +1)

**Validität:** die Gültigkeit einer Messung, d.h. ob überhaupt das gemessen wurde, was gemessen werden sollte (-1, 0, +1)

**Publikationsverzerrung:** 3 - keine Publikationsverzerrung; Gütekriterien einwandfrei

-1 – 2 - geringe Publikationsverzerrung; Gütekriterien größtenteils valide

< - 1 - Publikationsverzerrung gegeben

Basierend auf dem Mixed Methods Appraisal Tool (MMAT) ergänzt durch Angaben zu Gütekriterien und Publikationsverzerrung.
